# Anuric Acute Kidney Injury in a Patient with Left Flank Pain

**DOI:** 10.34067/KID.0000000000000114

**Published:** 2023-07-27

**Authors:** Andreia F. Curto, Anna E. Lima, Miguel Verdelho

**Affiliations:** Department of Nephrology, Hospital Professor Doutor Fernando Fonseca, Amadora, Portugal

**Keywords:** acute kidney failure, dialysis, ischemic renal failure, renal ischemia

## Abstract

**Figure:**
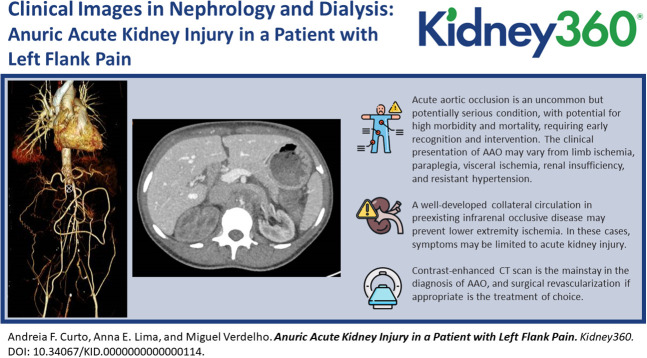

## Case Description

**Figure 1:**
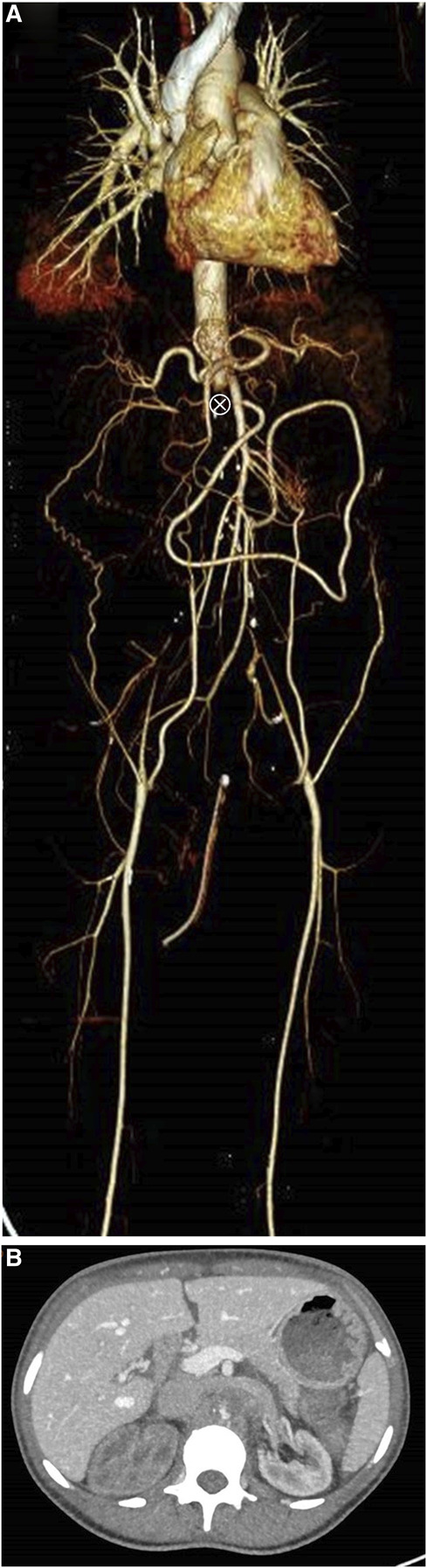
**Contrast-enhanced CT scan (3D reconstruction) showing acute aortic occlusion juxta-superior mesenteric artery.** (A) Aortic occlusion juxta-superior to the mesenteric artery (marked with an ⊗) along with collateral vessels in the abdominal wall on contrast-enhanced CT scan with 3D reconstruction. (B) A left kidney with contrast enhancement and right kidney without enhancement suggesting lack of kidney perfusion. CT, computed tomography.

A 48-year-old woman with a medical history of hypertension, chronic obstructive pulmonary disease, and active smoking presented to the emergency room with acute onset of right flank pain and acute kidney injury (serum creatinine [sCr] of 1.7 mg/dl) and increased serum inflammatory markers and pyuria. Upper urinary tract infection was considered, antibiotic treatment was started, and the patient was discharged the same day. One week later, the patient returned with acute left flank pain, without urinary symptoms. Vital signs were normal, except for high blood pressure. Physical examination was unremarkable, with no signs of peripheral hypoperfusion. Blood tests revealed deterioration of kidney function (sCr 6.9 mg/dl and BUN 66 mg/dl) and inflammatory markers persistently increased (leukocytes 28.100/mm^3^, C-reactive protein 12.9 mg/dl) with normal lactate dehydrogenase levels. Urine dipstick presented significant proteinuria, hemoglobin, and leukocytes. Upper urinary tract infection was diagnosed, and intravenous antibiotics were started. However, while performing the initial work-up, sudden anuria developed. A kidney Doppler ultrasound showed normal echogenicity and kidney size along with the signs of aortic occlusion juxta-superior mesenteric artery emergence, which was confirmed by contrast-enhanced computed tomography (CT) scan (Figure 1A). The CT scan also showed collateral circulation in the abdominal wall allowing sufficient blood supply to the lower limbs. Only the left kidney showed residual contrast uptake (Figure 1B) suggesting lack of perfusion of the right kidney. Emergent aortobifemoral bypass and end-to-side anastomosis of left renal artery and bypass graft were performed assuming that the left kidney was still viable. The patient started hemodialysis after surgery. Urinary output gradually increased along with kidney function recovery two weeks after surgery. These findings were supported by Doppler ultrasound showing normal flow in the left kidney (data not shown). Hemodialysis was withheld 27 days after surgery, and 3 months later, sCr was 1.7 mg/dl.

## Discussion

Acute aortic occlusion (AAO) is a life-threatening condition. The reported incidence of AAO is 3.8 per one million person-years, with no sex preponderance, and 30-day mortality rates between 21% and 52%.^1^

The clinical presentation of AAO may vary from limb ischemia, paraplegia, visceral ischemia, acute kidney injury, and refractory hypertension. The main cause of AAO is superimposed thrombosis of an atherosclerotic abdominal aorta. Other causes were excluded in this patient during follow-up. The most common location of AAO is infrarenal.^2^

A well-developed collateral circulation in preexisting infrarenal occlusive disease may prevent lower extremity ischemia and limit symptoms to acute kidney injury. Collateral circulation in our patient suggested a chronic aortoiliac atherosclerotic obstructive disease, with sudden progression of thrombus to the suprarenal level leading to acute renal failure and anuria. Active smoking is a well-established risk factor for atherosclerotic disease.^3^

Currently, contrast-enhanced CT scan is the mainstay in the diagnosis of AAO and can also reveal aortic dissections or aneurysms as the cause of occlusion, also aiding in surgical management. Doppler ultrasonography can be routinely used in the emergency room. Surgical revascularization is the treatment of choice, and options include retrograde transfemoral embolectomy, direct aortotomy, or extra anatomic bypass.^1,3^ This case highlights the importance of prompt diagnosis leading to a successful kidney recovery.

## Teaching Points

- Acute aortic occlusion is an uncommon but potentially serious condition, with potential for high morbidity and mortality, requiring early recognition and intervention. The clinical presentation of AAO may vary from limb ischemia, paraplegia, visceral ischemia, renal insufficiency, and resistant hypertension.
- A well-developed collateral circulation in preexisting infrarenal occlusive disease may prevent lower extremity ischemia. In these cases, symptoms may be limited to acute kidney injury.
- Contrast-enhanced CT scan is the mainstay in the diagnosis of AAO, and surgical revascularization if appropriate is the treatment of choice.
